# Recovery from Intracranial Hemorrhage Due to Leptospirosis

**DOI:** 10.1155/2011/504308

**Published:** 2011-10-05

**Authors:** Farhang Babamahmoodi, Abdolreza Babamhmoodi

**Affiliations:** ^1^Infectious Diseases Department, Mazandaran University of Medical Sciences, Sari, Iran; ^2^Health Research Center, Baqyiatallah University of Medical Sciences, Tehran, Iran

## Abstract

Intracranial hemorrhage is a rare and fatal presentation of leptospirosis. In this paper we present the case of a 51-year-old male farmer who lives in northern Iran. He came to our hospital with a severe headache. A paraclinical evaluation showed clear signs of thrombocytopenia, and a brain MRI revealed left temporoparietal hemorrhage. Our preliminary diagnosis was Leptospirosis, and after 26 days of hospital care the patient was discharged in good condition. This paper will educationally help physicians in better diagnosis and treatment of leptospirosis.

## 1. Introduction

Leptospirosis is one of the most common zoonotic infections around the world. Each year 1,500 to 2,000 cases are reported globally, but the real incidence is higher [1] because of clinical and laboratory limitation diagnosing the disease. The disease presents with variable combinations of clinical syndromes, which cause diagnostic confusion with many infectious and noninfectious diseases. Diagnostic problems have been presented in neuroleptospirosis. When a patient is infected with leptospirosis, the central nervous system is affected by aseptic meningitis or encephalitis (more rarely), and the peripheral nervous system suffers from neuritis, polyneuritis, and polyradiculoneuritis [2–4]. Other clinicalforms of neuroleptospirosis are intracranial hemorrhages, cerebellitis, and myelitis [1, 5]. In most cases, hemorrhage due to leptospirosis occurs in the pulmonary and gastrointestinal systems but not in the central nervous system [6]. In this paper we present a rare case of leptospirosis (Weil's disease) in a patient who came to our center (Razi Hospital in Ghaemshar city, Mazandaran province, Iran). In the patient's laboratory data we observed renal function impairment, increased liver enzymes, and a decreased platelet count (5,000).

## 2. Case Presentation

The patient is a 51-year-old man who arrived at our facility complaining of fever, chills, myalgia, and generalized weakness that had begun 5 days before coming to the hospital. His fever was continuous, and he reported that his eyes have been yellow for the previous 3 days; he also complained of a headache in the frontal area. He is a fishery officer and works in the rice paddies as well. He reported severe vomiting 4 to 5 times per day and a decreased appetite and a change in urine color during the previous few days.

The patient had no significant familial history of any disease. Except for one capsule of Omeprazole (20 mg) each day for heartburn; he reported no other current medications and no history of drug abuse history. The patient's physical examination showed the following vital signs: blood pressure 120/80 mmHG, pulse of 80, respiratory rate of 18, and temperature of 38°C. 

His conjunctiva was congested. No neck stiffness was detected. On both arms multiple petechia and, purpura were observed, but there was no sign of ecchymosis. The heart beat was normal. No organomegaly was detected in an abdominal examination. The lower limbs had normal appearance and function. 

The laboratory data are shown in Table 1.

**Figure 1:**
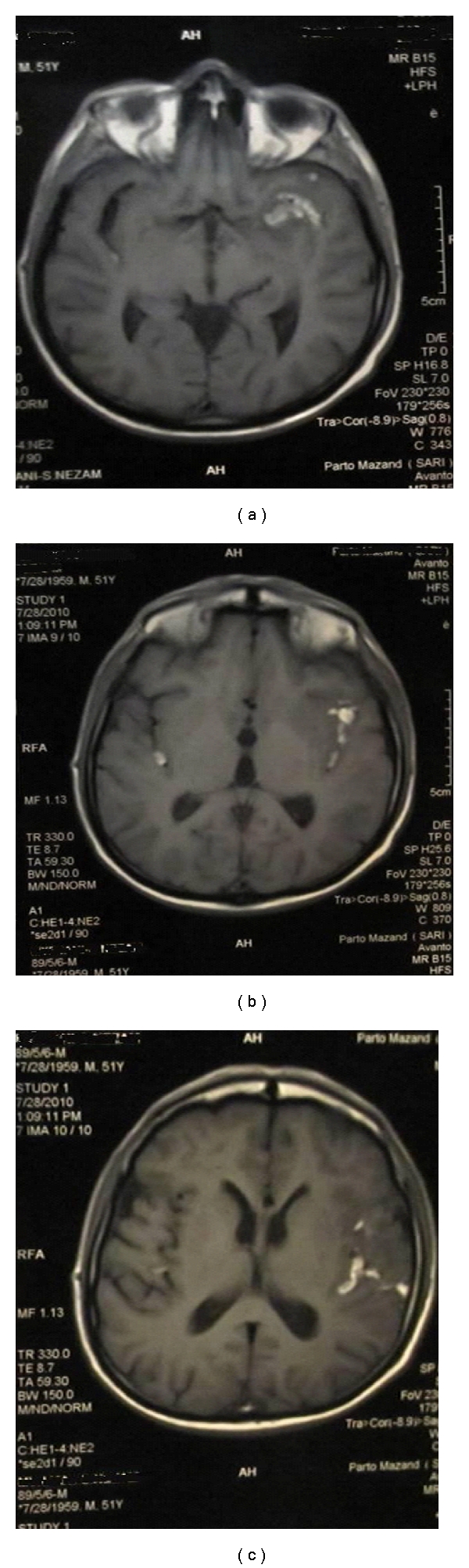
The patient's CT scan.

After a brain CT scan the radiologist reported “multiple hemorrhagic lesions in the temporal and parietal lobes, especially in the left side” (see Figure 1).

The patient's chest X-ray was normal, and he tested negative for HBs Ag and HBs AB.

His diagnosis was reviewed, and in view of the deranged hepatorenal functions and seasonal prevalence (recent floods in the area where the patient resided), leptospirosis was clinically suspected, which was further confirmed by the positive serum IgM-ELISA (IgG value of 100 and an IgM value of 20). Epidemics of leptospirosis in our region and unavailability of Microscopic agglutination test (MAT) made us to consider IgM-ELISA for confirmation of leptospirosis. 

In an abdominal sonography, 70 cc of fluid were found in Morison's pouch, and the liver exhibited hepatitis-like changes. An MRI showed intracranial hemorrhage, especially in the left lobe.

With intracranial hemorrhage care and Ceftriaxone (1 mg/day) and Dexamethasone (8 mg two times daily) for 1 week and 15 unit platelets, his condition improved. After 26 days of hospital care with a normal platelet count and normal renal function the patient was discharged from hospital.

## 3. Discussion

A study of 59 patients admitted to the Philippine General Hospital in July to November 1995 and June to October1996 showed that a prevalence rate of 61% for thrombocytopenia and its presence seem to indicate a more severe form of the disease. Most cases of thrombocytopenia are of moderate severity, and Casiple's findings revealed increased bleeding complications in the thrombocytopenic group. Mortality was also higher in the thrombocytopenic group. The majority of mortalities in this sample were associated with hemorrhagic causes, the most common of which was pulmonary hemorrhage (42%) [7].

Hospitalized patients often present with thrombocytopenia, hemorrhagic symptoms, jaundice, and renal failure. Patients in this situation usually die from septic shock complicated by multiorgan failure or a bleeding diathesis [8]. Pathological findings have revealed widespread hemorrhaging at mucosal surfaces, muscles, peritoneum, and various organs such as the heart, lungs, and kidneys in thrombocytopenic patients [9, 10]. In general, thrombocytopenia is frequently observed and associated with poor outcomes [11, 12].

A small proportion of patients develop severe icteric illness with renal failure. Jaundice occurs between the fourth and the sixth day but may occur as early as the second day or as late as the second to third week. The liver is often enlarged and tender. Jaundice is due to hepatocellular necrosis, intrahepatic cholestasis, and increased bilirubin load from absorption of tissue hemorrhage. Marked elevations of bilirubin with mildly elevated transaminases are some characteristic features of leptospirosis [13]. Neurological involvement in leptospirosis can present as meningitis, aseptic encephalitis, inflammatory myelopathy, Guillain-Barre syndrome, and stroke. Intracranial hemorrhage due to severe thrombocytopenia is a rare complication of Weil's syndrome [14–16].

In leptospirosis, as in other infectious diseases, the bleeding tendency is the result of imbalance in the haemostatic equilibrium, although it is unclear how this imbalance is triggered and what inflammatory and coagulation proteins are involved [17].

Panaphut and colleagues reported that “Ceftriaxone and sodium penicillin G were equally effective for the treatment of severe leptospirosis. Once-daily administration and the extended spectrum of Ceftriaxone against bacteria provide additional benefits over intravenous penicillin.” We also use Ceftriaxone for treatment of severe case, and so far we hade satisfaction from results [18]. 

Leptospirosis is one of the world's most prevalent zoonoses, with a clinical picture varying from a mild to potentially life-threatening disease in which haemostatic derangements play a central role. Despite these facts, leptospirosis is a neglected disease, which explains why many crucial aspects concerning its pathogenesis remain unanswered. Besides proper diagnosis and antibiotic therapy, which is the cornerstone of treatment, we urgently need to improve supportive treatment, especially for cases with life-threatening bleeding complications.

## Figures and Tables

**Table 1:** Laboratory data.

Test	Patient's data	Normal value	SI units
WBC	14100/mcL	3800–9800/mcL	3.8–9.8 × 10^9^/L
RBC	3530000/mcL	4.3–5.9 × 10^6^/mcL	4.3–5.9 × 10^12^/L
PLT	5000/mcL	150–450 × 10^3^/mcL	150–450 × 10^9^/L
HgB	10.6 gr/dL	13.8–17.5 g/dL	138–175 g/L
Creatinine	2.8 g/day	0.8–1.8 g/day	7.1–15.9 mmol/day
FBS	117 mg/dL	65–115 mg/dL	3.6–6.3 mmol/L
CK	2990 ng/mL	174–320 ng/mL	0.5–3.67 mckat/L
Amylase	350 IU/L	35–118 IU/L	0.58–1.97 mckat/L
LDH	900 IU/L	100–250 IU/L	1.67–4.17 mckat/L
INR	1.0	0.9–1.2	0.9–1.2
AST	395 IU/L	11–47 IU/L	0.18–0.78 mckat/L
ALT	250 IU/L	7–53 IU/L	0.12–0.88 mckat/L
ALP	160 IU/L	20–130 IU/L	20–130 IU/L
PT	12.5 s	10–14 s	10–14 s
PTT	42 s	32–45 s	32–45 s
ESR	98 mm/hr	≤30 mm/hr	≤30 mm/hr
BUN	159 mg/dL	8–25 mg/dL	2.9–8.9 mmol/L
RBC in Urine Analysis	14-15/HPF	0–2/HPF	0–2/HPF
Total bilirubin	49 mg/dL	0.1–1 mg/dL	2–18 mcmol/L
Direct bilirubin	45.3 mg/dL	0.1–1 mg/dL	2–18 mcmol/L
